# Family systems nursing conversations: influences on families with stroke

**DOI:** 10.1186/s12912-022-00873-7

**Published:** 2022-05-06

**Authors:** Susanna Pusa, Britt-Inger Saveman, Karin Sundin

**Affiliations:** 1 Department of Nursing, Umeå University, Campus Örnsköldsvik, Box 843, S-891 18 Örnsköldsvik, Sweden; 2 Department of Nursing, Umea University, S-901 87 Umeå, Sweden

**Keywords:** Family conversations, Family health, Family nursing, Family systems nursing, Stroke, Transition theory

## Abstract

**Background:**

Since a family member’s stroke affects the entire family, family systems nursing conversations (FSNCs) may be an appropriate intervention to support the family as a whole. The purpose of our study was to illuminate family members’ experiences within their family situations 6 months after participating in FSNCs when a family member under 65 years of age had suffered a stroke.

**Methods:**

Fourteen semi-structured follow-up interviews were conducted with family members 6 months after they had completed a series of 3 FSNCs. The interview transcripts were subjected to qualitative content analysis.

**Results:**

Family members experienced that the FSNCs had contributed to greater understanding of each other and greater closeness in the family. The FSNCs had also facilitated a mutual understanding of the family’s situation, which they could better manage and move forward with together.

**Conclusions:**

FSNCs can support relational aspects and healthy transitions within families. However, long-term follow-up research is needed to generate sound evidence and inform education about FSNCs, as well as to facilitate their implementation. As a result, families may become better able to prevent the negative outcomes of illness in the family.

## Background

When a family member suffers an illness, such as a stroke, the entire family is affected. However, the ways that families manage the situation can vary depending on the family’s prior situation [1]. Since many stroke survivors face significant challenges, such as pain, hemiparesis, difficulty swallowing, visual impairment, fatigue, communication difficulties and cognitive and emotional changes [2], family members often become their natural support system. A prolonged process may be required to organise the responsibilities of providing care within the family before order and stability are regained, and solutions for managing everyday life have been found. Striking a new balance, adapting to new routines and integrating the changes caused by stroke can be challenging for families. Although they require increased support and understanding from healthcare professionals and close family friends [3, 4] during the acute phase and long after [5, 6], long-term support for families seldom occurs [6, 7]. As a consequence, feelings of loneliness and being unsupported can intensify, as reported by families [7] up to 7 years after a family member’s severe traumatic brain injury. Such long-term experiences attest to the importance of providing support to families in healthcare settings.

In recent decades, interest in family health in relation to stroke care has grown, with stroke being increasingly recognised as a family matter [8]. Nurses working in stroke care assess both the patient’s and the family’s needs as a means of providing tailored care and support to the family as a whole [9]. A recent scoping review of the interventions used to support families of survivors of brain injury, such as stroke, revealed that family-oriented interventions are common and most often prioritise emotional support as their core component [8]. In the context of stroke, emotional support, described as a caring interaction [10], can support survivors and their families in gaining insights into their situations from new perspectives [11].

Within healthcare, families can be treated in different ways depending on the healthcare professionals’ perceptions. For example, although not a widely influential framework in nursing practice or research, family systems nursing views the family as a unit and a system. It assumes that a set of human relationships can be likened to a system in which all parts interact and the whole is more than the sum of its parts. Following the family systems nursing framework, one way to support the family of someone who has suffered a stroke is to host family systems nursing conversations (FSNCs). These are nurse-led family conversations with a family systems approach [1, 12] whereby relationships between family members, and between nurses and families, have to be nurtured and maintained in order to create an environment for change [13]. Similarly, the approach emphasises that nurses view the family as a system, with special focus on relationships and interactions between family members, since the family needs support as a whole [1, 12].

Along with mounting clinical evidence suggesting that families benefit from the support of FSNCs [13, 14], nurse-led family conversations based on or inspired by the Calgary models [1, 12] are increasingly used in North America, Asia, and Europe [15, 16]. The Calgary models involve the Calgary Family Assessment Model (CFAM), the Calgary Family Intervention Model (CFIM) [1], and the Illness Belief Model [12]. These models are multidimensional, enabling nurses to assess family strengths, resources, difficulties and illness suffering. This is done through targeted questions connected to structural, developmental and functional aspects (CFAM). They also provide a framework for nursing conversation intervention by nurses to families (CFIM) [1]. The illness belief model has an expanded focus on family health connected to the illness beliefs of patients, families, and healthcare professions [12].

An integrated literature review revealed that family members with various health problems (e.g. brain disease, heart disease, cancer, and psychiatric diseases) experienced mostly positive outcomes from FSNCs in the cognitive, affective, and behavioural domains of family functioning [17]. Since the review, additional studies have shown promising results regarding FSNCs for family well-being, including for example from Sweden [11, 18–21], the Netherlands [22], Taiwan [23], Iceland [24], Denmark [25], Thailand [26], and Canada [27]. All these studies have contributed to clinical evidence indicating that FSNCs outperform conventional care in providing social support [25], reducing the burden on family caregivers, and improving family functioning [22]. In addition, FSNCs can improve the emotional and cognitive support of family caregivers [28].

However, research on the long-term outcomes of FSNCs [22, 25] is limited, especially concerning families of those who have suffered a stroke [11]. The purpose of our study was, therefore, to illuminate family members’ experiences within their family situations 6 months after participating in FSNCs when a family member under 65 years of age had suffered a stroke.

## Method

### Study design

Using an inductive design with qualitative content analysis, our follow-up study sought to determine whether the intervention of FSNCs had facilitated change within families where a member had suffered a stroke. It also explored whether the conversations had helped families perceive new ideas, meanings, beliefs, and/or opportunities that could assist them in solving family problems.

### Participant recruitment

Recruitment began when a family member who had suffered a stroke was hospitalised at either of two rehabilitation centres in northern Sweden. After receiving permission to conduct the study, a nurse from each unit in the centres issued a call for families to participate. The nurses supported recruitment by identifying families who met the inclusion criterion, primarily having a family member under 65 years of age who had suffered a stroke. The nurse asked persons who had suffered a stroke, did not have severe aphasia and could communicate in Swedish whether they were willing to participate in the study. If interested, they received verbal and written information about the study and, if they agreed, provided their names and contact information. A member of the research group then contacted each prospective participant to give more detailed information about the study and request participation. Those agreeing signed a written consent form and were asked to state who they considered to be family members and could join them in the FSNCs and engage in a follow-up interview. In total, 17 family members, including the individuals who had suffered a stroke, from seven families participated in the intervention. Family members under 15 years of age were not included since it was assumed that only then can adolescents usually understand and give informed consent, and are sufficiently mature to participate and understand the import of the FSNCs and the follow-up interview.

Each intervention was held in the family’s home between 4 and 7 months after the stroke and led by two registered nurses in the research group specially trained in family systems nursing. Structured around the core components of FSNCs [14], and based on the Calgary family assessment model, the Calgary family intervention model [1], and the illness belief model [12], the intervention entailed a series of three FSNCs lasting 40–60 min each held at approximately 2-week intervals [29]. Two weeks after the third FSNC [30], a closing letter was sent to each family.

### Data collection

In the individual 6-month follow-up interview (presented in this study) 14 of 17 family members participated. The family members who did not participate were two stroke survivors and an adolescent. Table 1 presents the demographic data of the 14 family members who were interviewed.

**Table 1:** Participants in the follow-up interviews (*n* = 14)

**Family members**	Person with stroke	5
	Spouse, partner, or cohabiter	5
	Adolescent > 15 years old	4
**Gender**	Persons with stroke: men/women	5/0
	Relatives: men/women	3/6
**Age**	Persons with stroke: median (range)	58(49–64)
**Diagnosis**	Infarction or haemorrhage	5/0

The same interview guide was used for all follow-up interviews and based on the theoretical starting point for FSNCs as developed in Sweden [29], the Calgary family assessment model, the Calgary family intervention model [1], and the illness belief model [12]. The interviews were conducted during 2013 independently by a member of the research group who had not previously met the families. The family members were asked whether they perceived that the three FSNCs had influenced them and, if so, how. Other examples of questions were “Could you tell me in what ways you experienced the conversations to be helpful for your family?” “Has there been any change in your family situation?” and “Could you tell me whether you and your family members contributed to the conversation and, if so, in what ways?” The interviews were conducted in the families’ homes, lasted 20–60 min, and were audiotaped and transcribed verbatim. The interview transcripts were coded to anonymise the data and the codes kept separate from the text. No individuals can be identified in the quotations presented here.

All authors are registered nurses with a doctoral degree and have various clinical work experience (including caring for persons who have suffered a stroke). They belong to a research group studying family systems nursing.

### Data analysis

The *unit of analysis* was the interview transcripts. These were considered as a single text and subjected to qualitative content analysis [31–33] based on an inductive approach. No specific theory was applied in interpreting the text, nor were the researchers’ pre-understandings in the forefront during the analysis. The text was read several times by all authors to gain an understanding of the content as a whole. The text was then broken down into meaning units related to the purpose of the study. A *meaning unit* was defined as a meaningful part of the text, linked in content and context, and could consist of words, sentences or paragraphs. The meaning units were then *condensed* in a process that shortened sentences without sacrificing the core content. The condensed meaning units were given *codes* at a higher level of abstraction, although if possible at the same level of abstraction, while still describing the content. The codes were then compared for differences and similarities and sorted into essentially mutually exclusive *subcategories*. Each subcategory consisted of several codes with at least one common feature, and the subcategories were later sorted into *categories*. The last author performed the primary analysis (i.e. identified meaning units, assigned codes, and proposed subcategories and categories). All authors then discussed and agreed upon the relevance and internal consistency of the codes and subcategories by alternately reviewing the text, codes and subcategories. Ultimately, consensus about the categorisation and the naming of the categories was reached, which ensured the trustworthiness and credibility of the analysis. Categories and subcategories are described in the results with excerpts from the text to promote internal consistency (cf. [31–33]). The Standards for Reporting Qualitative Research [34] were consulted while developing the report of our results.

## Results

The results, organised in two categories and five subcategories, are shown in Table 2.

**Table 2:** Overview of categories and subcategories

**Category**	**Subcategories**
**Improved closeness between family members**	Enhanced understanding of oneself and other family members
	Enhanced family bonds
**Renewed mutual comprehensive understanding**	Development of a shared understanding
	Shared management of the family situation
	Moving forward

### Improved closeness between family members

The first category captures the process of becoming closer to the other family members. Since the family situations were influenced by an improved understanding of both the other family members’ views and beliefs and their own, a more open dialogue also improved the relationship. Feelings of becoming closer to other family members enhanced the family bonds.

#### Enhanced understanding of oneself and other family members

Family members perceived that the structured family conversations had initiated a process that helped them to talk with each other more often. Having the opportunity to talk without being interrupted, hearing what other family members thought, sitting together, and actively listening to each other were all viewed as benefiting mutual understanding. Moreover, the experience of each person being equally focused and given equal space was considered positive. The family members especially valued the opportunity to tell their stories and to be listened to during family conversations, both of which contributed to feelings of being visible and validated. By sharing thoughts and feelings, the family members experienced that their own beliefs became more apparent to themselves and they better understood the beliefs of other member concerning the family’s situation.

*“I could almost step aside and look at myself and look at, start analysing, and reflecting on the people around me.”* (C1)

#### Enhanced family bonds

The family members felt that the FSNCs had made them more open and helped them to see their own and other members’ strengths and vulnerabilities. Through this they had become closer and bonded in stronger ways than before, and the relationships and cohesion between family members had also been enhanced. The family conversations were valued for illuminating dimensions of the relationships and experiences of all family members that had not been considered earlier at the rehabilitation centre. Being invited to the conversations as an in-group member also created a sense of being equally important to the family’s well-being as every other member. In addition, the family members described beginning to engage in activities together that they had not done before.

*“The relationship between my relative and me has become stronger. That’s how I’ve experienced it. And you could say that the conversations contributed to that. I absolutely think so.”* (G2)

### Renewed mutual comprehensive understanding

The second category captures how the family situation could be viewed with a renewed comprehensive understanding of the family as a unit. It encompasses the family’s joint perspectives on, and activities towards, a shared understanding, managing the family situation, and envisaging the future.

#### Development of a shared understanding

By sitting together and talking about their feelings and thoughts, the FSNCs had given the family members a clearer picture of what the stroke had meant for the family as a unit. By reflecting on and gathering their own and other family members’ beliefs and experiences, they could form a kind of shared family understanding. However, the enhanced mutual understanding did not necessarily mean that the family members had the same thoughts. On the contrary, it involved developing a clearer overall picture of the family situation, which had increased the sensitivity to different perspectives within the family.

*“But I still get the feeling that you receive more attention and an enhanced understanding when everyone hears how everyone else experienced it [the stroke].”* (D1)

#### Shared management of the family situation

The family members experienced that the FSNCs had catalysed a process that helped them to manage the family situation by together analysing and reflecting on what had happened and what could happen in the future. The family conversations had prompted the family members to dare to start talking with each other in new ways. By sharing their experiences, such as hopes and fears, they also started to view the family situation differently and to recognise hidden beliefs, thoughts and insights. Consequently, the family members formed a more realistic view of their family situation and gained the strength to be able to manage the situation together.

*“When we talked and when we listened to each other, a lot of it wasn’t new, but we still moved forward. It was like a process.”* (A3)

#### Moving forward

The family members also felt that the FSNCs had made moving on easier. The family conversations were found to have broadened perspectives related to the family’s situation and future. They began to see themselves and the other family members from a new perspective, with new ways of thinking and a more positive belief in the present situation and the future. The family members had also learned, for future reference, to share their thoughts and experiences with each other in ways not previously used. Through the FSNCs they had acquired new tools to handle the family situation in the future.

*“There may be some hard topics that you don’t bring up with others around you, but you can do it here [during the FSNCs]. So, then it becomes as one says: like an opening to use on the way ahead.”* (E2)

## Discussion

The purpose of our study was to illuminate family members’ experiences within their family situation 6 months after participating in FSNCs when a family member under the age of 65 years had suffered a stroke. The participants reported that the family conversations had brought the family members closer together and allowed them to understand each other and the situation in a more comprehensive way; this had enabled them to manage the situation and to move forward.

The findings of our study indicated that, after 6 months, the FSNCs had supported the family members in talking together and reflecting on challenges and aspects of their family situation that had previously gone unmentioned or were difficult to discuss. In particular, the family members felt included by being invited to the conversations and having an opportunity to enhance their understanding of themselves and each other. Similarly, a study focusing on young families with stroke [35] found that supportive family conversations can contribute to an empathetic understanding of the experience. This can be valuable because family members of stroke survivors can experience feeling left behind and disconnected amidst interrupted family processes [36, 37]. Since our follow-up study showed that FSNCs can enable family members to feel closer to each other, strong relationships within the family need to be supported and facilitated [11, 36]. This is consistent with the results of a review [38] showing that relatives need opportunities for counselling as a means of supporting reflection and developing strategies for coping with the transition at home after their loved one is discharged from inpatient stroke rehabilitation.

Even 6 months after the intervention with FSNCs, the participants expressed how the conversations had provoked new thoughts by giving them the opportunity to express themselves. The conversations therefore seem to have induced a process that helped them move on and renew their comprehensive understanding of the family situation. The same outcome was also evident in a comparable study [39] in which family members found FSNCs meaningful and helpful. By telling their stories and listening to others’ experiences, the family members began to think differently and see new meaning and possibilities in relation to their family situations. Receiving answers was not important, but rather that they had been helped to think from alternative perspectives and that others had listened to them (cf. [39]). Other research has shown that family conversations empowered participants to see things differently and gain another perspective on their life situation [40]. A further study [41] revealed that family conversations contributed to giving family members a more positive attitude and increased confidence in their inner strength to better manage family situations.

We also found that the FSNCs had helped the family members move on by enabling them to identify and develop solutions to their problems. This finding aligns with past results showing that listening to each other in the family increased mutual understanding and resulted in empowering and affirming behaviour [18, 40, 42]. Several other studies have underscored the strong healing effect of FSNCs [19, 43, 44], both on participants as individuals and at family level [40].

A model of the benefits and working mechanisms of FSNC was created to identify and illustrate their value for families during and immediately after the conversations, and in the long term [45]. Findings underscore that benefits, such as reducing a perceived caregiver burden and improved quality of care for patients, gradually manifested in the weeks after the FSNCs. Consequently, the families perceived benefits in their current situations and a shared starting point for the future [45]. From the findings in our study, we can add that the health-promoting process may have continued even after 6 months.

FSNCs supporting healthy transitions from an initial period of distress towards experiences of enhanced stability and balance in the family situation can be clarified by applying the Transition Theory developed by Afaf Meleis [46]. Positive indicators of healthy transitions include feeling connected, interacting with others, being situated, developing confidence, and coping [46]. All of these relate to our findings that enhanced family relationships with deepened connections facilitated strengthened bonds and family cohesion, leading to cooperation within the family. Furthermore, dialogue within the family improved, initiating a process of including each other and talking more openly. When family members were able to narrate their stories, an increased awareness of their own and others’ beliefs and experiences about the situation became visible. Such awareness can be understood as promoting interaction [46], so that the meaning of the transition and behaviours in response can be revealed, clarified and acknowledged.

The family members in our study stated that the FSNCs had supported them in finding common ground where reflection on the past, present, and future was emphasised. In turn, they began viewing their situations differently following reformulations of their previous thoughts and insights. Being situated, therefore, involved creating new meanings and perceptions (cf. [46]). The family members also expressed enhanced confidence and a sense of security in sharing and talking about challenging aspects of the family situation with each other. This outcome could be linked to their experiences with, and development of, confidence and coping, two dimensions of the transition process that refer to resource utilisation and development of strategies for managing and manifesting confidence (cf. [46]). The stronger relationships marked by enhanced interaction and understanding between family members due to FSNCs in our study could be understood as supporting healthy transitions.

The interaction between the family and the nurse during the FSNCs could be linked to the way transitions occur within the context of several relationships. These can include the multifaceted, dynamic relationships between patients, family members, and nurses (cf. [47]). Such relationships influence the emergence of the transition process, self-organisation, and deriving meaning from the process. Thus, an environment is needed in which healthcare professionals facilitate supportive communication and dialogue between a family member with a chronic disease, their family members and a nurse. Health-promoting processes and results occur as a result of the interaction between all of those parties. Professional caregivers, such as nurses, should therefore not only focus on executing tasks but on simultaneously actively developing and deepening their relationships with families as units (cf. [47]).

Our results 6 months post-intervention – family members’ becoming closer and experiencing renewed understanding – can be seen as aspects of improved family functioning. This aligns with the findings of another follow-up study 6-months after FSNCs [22], which showed that family functioning improved for patients and family members in the intervention group compared with the control group. Furthermore, the results revealed that FSNCs had reduced the family caregiver burden. In contrast, another randomised follow-up study [25] using FSNCs as an intervention showed no differences between the intervention and control groups from scores on family health and family functioning scales amongst participating patients and family members; however, the participants did report improved social support. These mixed results highlight the importance of conducting additional follow-up studies to strengthen evidence that FSNCs do indeed impact family health and family functioning.

In addition, we propose studies focused on implementing FSNCs as a way to facilitate the incorporation of family systems nursing in clinical settings. In recent years, studies oriented towards its implementation have increased [48–51]; however, more research is needed to understand the complex process of implementation of family systems nursing [51] in order to improve care for both patients and their families.

### Strengths and limitations

In qualitative studies, the sample size depends on the aim of the study and the quality of the data [31]. In our follow-up interviews, the sample of 14 family members included all but three individuals who participated in the intervention (i.e. the FSNCs). The data were detailed, rich in content, and thus considered to be sufficient to fulfil the study’s purpose. The gender distribution in the overall sample was evenly spread, with eight men and six women, although all the men were stroke survivors.

All the researchers sought to minimise the influence of their pre-understanding during the interviews and data analysis. However, during the inductive analysis the risk of bias remained since the research topic being studied was known in advance.

Even though the family members clearly described the intervention as positively influencing their family situation and transition, some of the outcomes could have been related to circumstantial factors (such as external events or developments) arising in the 6 months between the third FSNC and the follow-up interview.

Regarding transferability, our findings are likely relevant to other similar interventions involving health-promoting FSNCs.

## Conclusions

Our results from a follow-up study 6 months after a series of 3 FSNCs lend credence to evidence that FSNCs constitute a sustainable method for supporting relational and emotional aspects within families, and enhancing health and family functioning following stroke in the family. When a family member suffers a stroke, the ensuing process can be understood as a transition in which nurses can play a key role in addressing the family according to a comprehensive relational approach. However, follow-up research is needed to clarify other long-term effects of FSNCs as a means of systematically gathering sound evidence from a relational perspective. Education and implementation focused on how nurses, together with families, can use existing resources to manage illness and its consequences by supporting transitions towards healthier lives are also required. Such nurse-led conversations with all family members may improve the ways by which families can prevent the negative outcomes of illness.

## Data Availability

The data generated in our research and its analysis are not publicly available. In accordance with ethical principles concerning confidentiality observed by the Swedish Research Council [52] unauthorised individuals may not access our research data, which includes original data from interviews. To ensure privileged access only by the authors, all research data are stored in a safe deposit box at the university.

## Abbreviations

CFAM: Calgary Family Assessment Model
CFIM: Calgary Family Intervention Model
FSNC: Family systems nursing conversation
